# Long-term outcomes of childhood sexual abuse: an umbrella review

**DOI:** 10.1016/S2215-0366(19)30286-X

**Published:** 2019-09-10

**Authors:** Helen P Hailes, Rongqin Yu, Andrea Danese, Seena Fazel

**Affiliations:** Department of Psychiatry, University of Oxford, Oxford, UK; Medical Research Council Social, Genetic and Developmental Psychiatry Centre and Department of Child and Adolescent Psychiatry, Institute of Psychiatry, Psychology and Neuroscience, King’s College London, London, UK; National and Specialist CAMHS Trauma and Anxiety Clinic, South London and Maudsley NHS Foundation Trust, London, UK; Department of Psychiatry, University of Oxford, Oxford, UK

## Abstract

**Background:**

Although many meta-analyses have examined the association between childhood sexual abuse and subsequent outcomes, the scope, validity, and quality of this evidence has not been comprehensively assessed. We aimed to systematically review existing meta-analyses on a wide range of long-term psychiatric, psychosocial, and physical health outcomes of childhood sexual abuse, and evaluate the quality of the literature.

**Methods:**

In this umbrella review, we searched four databases (PsycINFO, PubMed, Cumulative Index to Nursing and Allied Health Literature, and Global Health) from inception to Dec 31, 2018, to identify meta-analyses of observational studies that examined the association between childhood sexual abuse (before 18 years of age) and long-term consequences (after 18 years). We compared odds ratios (ORs) across different outcomes. We also examined measures of quality, including heterogeneity between studies and evidence for publication bias. This study is registered with PROSPERO, CRD42016049701.

**Findings:**

We identified 19 meta-analyses that included 559 primary studies, covering 28 outcomes in 4 089 547 participants. Childhood sexual abuse was associated with 26 of 28 specific outcomes: specifically, six of eight adult psychiatric diagnoses (ORs ranged from 2·2 [95% CI 1·8–2·8] to 3·3 [2·2–4·8]), all studied negative psychosocial outcomes (ORs ranged from 1·2 [1·1–1·4] to 3·4 [2·3–4·8]), and all physical health conditions (ORs ranged from 1·4 [1·3–1·6] to 1·9 [1·4–2·8]). Strongest psychiatric associations with childhood sexual abuse were reported for conversion disorder (OR 3·3 [95% CI 2·2–4·8]), borderline personality disorder (2·9 [2·5–3·3]), anxiety (2·7 [2·5–2·8]), and depression (2·7 [2·4–3·0]). The systematic reviews for two psychiatric outcomes (post-traumatic stress disorder and schizophrenia) and one psychosocial outcome (substance misuse) met high quality standards. Quality was low for meta-analyses on borderline personality disorder and anxiety, and moderate for conversion disorder. Assuming causality, population attributable risk fractions for outcomes ranged from 1·7% (95% CI 0·7–3·3) for unprotected sexual intercourse to 14·4% (8·8–19·9) for conversion disorder.

**Interpretation:**

Although childhood sexual abuse was associated with a wide range of psychosocial and health outcomes, systematic reviews on only two psychiatric disorders (post-traumatic stress disorder and schizophrenia) and one psychosocial outcome (substance misuse) were of a high quality. Whether services should prioritise interventions that mitigate developing certain psychiatric disorders following childhood abuse requires further review. Higher-quality meta-analyses for specific outcomes and more empirical studies on the developmental pathways from childhood sexual abuse to later outcomes are necessary.

**Funding:**

Wellcome Trust.

## Introduction

Child sexual abuse is a global public health concern and is associated with a wide range of adverse outcomes. A meta-analysis of 217 publications has reported a global prevalence of 12% on the basis of 331 independent samples comprising about 10 million individuals.^1^ Studies have reported associations between childhood sexual abuse and many psychosocial and health-related outcomes, including psychosocial problems,^2,3^ self-harm,^4^ psychiatric disorders,^5–7^ and physical health diagnoses such as HIV^8^ and obesity.^9^ These reports have raised important questions about the extent of association between childhood sexual abuse and long-term outcomes in adulthood, the relative effects of childhood sexual abuse on different outcomes, and the quality of the current research base. For example, the long-term effects of sexual abuse on psychiatric outcomes might be stronger than that on physical health ones because of possible over-reporting of symptoms in individuals with psychiatric disorders.^10,11^

Previous attempts to synthesise the large number of meta-analytical reviews in the field have mostly considered solely mental health outcomes or a single outcome, such as depression, anxiety disorders, suicide and self-injury, or substance misuse.^12–15^ These reviews have been difficult to compare because their definitions of childhood sexual abuse have differed, they have included different types of study design, and the outcomes vary from diagnoses to self-report markers of morbidity. Furthermore, it has not been possible to identify research gaps in the field, and determine outcomes that are not synthesised or whether there are differential effects of childhood sexual abuse on outcomes. In addition, these reviews have usually been based on small sample sizes and searched literature that is typically more than a decade old, and lack quantitative synthesis and quality assessment.^12–17^ We aimed to address these limitations and provide an overview of the breadth and validity of the associations of childhood sexual abuse with a diverse range of long-term important psychosocial, psychiatric, and physical health outcomes. Our goal was to provide a comprehensive synthesis of the effects of childhood sexual abuse on morbidity and disability in adulthood, which could help to identify targets for clinical and policy interventions.

## Methods

### Search strategy and eligibility criteria

We did an umbrella review, in which information from existing meta-analyses of studies on outcomes of childhood sexual abuse was systematically collected and evaluated. We did a keyword search of titles and abstracts in four major digital databases, PsycINFO, PubMed, Cumulative Index to Nursing and Allied Health Literature, and Global Health, for papers published from database inception to Dec 31, 2018, with no language restriction. These databases included dissertation abstracts, and our search included unpublished grey studies. The same keywords were used for each database search for sexual abuse (“sexual abuse” OR “sexual assault” OR “sexual trauma” OR “sexual crime” OR “rape” OR “incest” OR “molestation” OR “victim*” OR “maltreatment”), childhood (“child*” OR “youth” OR “adolescent” OR “young” OR “teen*”), and meta-analysis (“meta-analy*” OR “meta-regression” OR “meta-synthesis”). To include a maximum number of eligible studies that examined long-term outcomes of childhood sexual abuse, we did not set any limit on the outcome in the primary systematic search. We did a secondary search in PsycINFO for the top ten global disability-adjusted life-year (DALY) risks,^18^ as well as for the top ten global mental and behavioural health DALYs (appendix p 1).^19^ The references of other umbrella reviews on outcomes of childhood sexual abuse were manually searched.^12–17^ We also used forward and backward citation chaining to supplement our search.

Inclusion criteria were meta-analyses that reported outcome data for childhood sexual abuse, disaggregated from other forms of abuse; in which the majority of outcomes were in adults (defined as average participant age at time of outcome measurement was older than 18 years for more than 70% of the included primary studies); in which the majority of participants were children at the time of abuse (defined as average participant age at time of abuse being younger than 18 years for more than 70% of the included primary studies); and that provided aggregate quantitative effect sizes for health or psychosocial outcomes (eg, odds ratio, Pearson’s *r*, or Cohen’s *d*).

When more than one meta-analysis reported data for the same outcome, the most recent review that met our inclusion criteria was selected, and older meta-analyses were excluded to avoid duplication of samples. When two or more meta-analyses reported data for the same outcome and were published within the same year, the meta-analysis including the greatest number of primary studies was selected, and the others were excluded.^20–27^ One study was unavailable (and author not contactable).^28^ In addition, we excluded studies only reporting prevalence of childhood sexual abuse in a selected sample with a certain outcome (eg, homelessness).^29^

Because data collection resulted in effect size statistics for nearly 100 different outcomes, they were systematically narrowed down using the following criteria: for health outcomes, specific diagnoses (eg, schizophrenia and fibromyalgia) and related symptoms (eg, psychosis and pain) were included, but subcategories of diagnosis (eg, social phobia as a subcategory of anxiety and anorexia nervosa as a subtype of eating disorder) and other symptoms (eg, cardiopulmonary symptoms and chronic pelvic pain) were excluded (appendix pp 2–3). Of psychosocial outcomes, the ten outcomes including the largest number of primary studies, ranging from seven to 45 primary studies, were included (eg, adult sexual revictimisation and substance misuse), whereas outcomes including fewer primary studies, ranging from two to six primary studies, were excluded (eg, recent unprotected anal intercourse, online sexual offending compared with offline sexual offending, self-esteem, and hostility). There was some duplication of participants between anxiety and anxiety symptomatology, and depression and depressive symptomatology. Therefore, the actual sample sizes might be slightly smaller. Because the odds ratio (OR) for sleep disorders (16·2 [95% CI 2·1–126·8]) was derived from a single primary study and had a large 95% CI, these data were excluded as an outlier. The minimum number of primary studies in the eligible meta-analytical reviews was three.

Data extraction was conducted following a predetermined data extraction form (appendix pp 4–5). Effect sizes were extracted from the most parsimonious model because not all studies provided adjusted effect sizes. Sources of heterogeneity, if examined, were reported. The initial screening for inclusion and exclusion of studies was conducted by HPH. A second extractor (SG) was involved in the extraction of effect sizes. In the case of any uncertainty in inclusion and exclusion of studies and data extraction, RY and SF were consulted and any conflicts were resolved through discussion between HPH, RY, and SF. A study protocol was registered with PROSPERO.^30^

### Data analysis

All effect sizes and CIs were converted into ORs to enable comparison across outcomes.^31,32^ That is, estimates of effects from each meta-review were converted into a common metric, ORs, representing the odds that an outcome (eg, suicide) would occur given a particular exposure (childhood sexual abuse) compared with the odds of the outcome occurring in the absence of that exposure. We calculated population attributable fractions for each outcome on the basis of the relative risk due to childhood sexual abuse and the proportion of the population exposed to childhood sexual abuse, assuming causality between exposure and outcomes.^33^ No confounder-adjusted effect size data were reported for outcomes, so we calculated population attributable fractions using the confounder-unadjusted attributable risk formula and the conservative 10% prevalence estimate of childhood sexual abuse.^34–36^

The AMSTAR (a measurement tool to assess systematic reviews) checklist was used to assess the methodological quality of the included systematic reviews,^37^ and some items were modified for this umbrella review (appendix pp 6–7). For all studies, one point was awarded for each of the eleven criteria met. Scores of 0–3 were considered low, scores of 4–7 were considered medium, and scores of 8–11 were considered high quality.^37^ HPH and RY did the quality assessment.

Quality was further assessed in the following ways. First, we measured heterogeneity, the relative inconsistency of studies pooled in a particular meta-analysis, using the *I*^2^ statistic.^38^ *I*^2^ is reported as a percentage, where scores of less than 50% were considered low heterogeneity.^39^ Second, we applied the excess significance test, a measure of publication bias that compares the expected versus the observed significance in a meta-analysis.^40^ Excess significance was calculated as a ratio of the overall OR of each meta-analysis to the OR of the largest primary study included in that meta-analysis, with a ratio greater than 1 indicating bias for publication of an excess number of significant results in the literature.^41^ To supplement the evaluation of excess significance, we also collected information on another marker of publication bias, small study effects, from the individual meta-analysis included. Third, when data on τ^2^ estimated heterogeneity and SE (standard error of the effect sizes) were available, prediction intervals were calculated. A 95% prediction interval gives a range of scores in which a future sample statistic can be said to fall with 95% certainty. If the prediction interval includes the null OR of 1, future studies might find that the exposure produced no effect or the opposite effect on the outcome.^42^

We created an overall quality assessment. Each outcome was assigned a score of 0 or 1 for the five categories of heterogeneity between studies (*I*^2^), publication bias (excess statistical significance and small study effects), prediction intervals, and AMSTAR quality measurement scores, with 0 representing low quality and 1 representing high. The five quality analysis scores were then summed to determine an aggregate quality score within the range of 0–5, with 0 designating the lowest overall quality and 5 designating the highest. Because this study is an umbrella review of systematic reviews, we did not assess the quality of individual studies included in each systematic review. ORs were presented in a forest plot using STATA, version 14.

This study is registered with PROSPERO, CRD42016049701.

### Role of the funding source

The funder of the study had no role in study design, data collection, data analysis, data interpretation, or writing of the report. HPH had full access to all the data in the study, and all authors had final responsibility for the decision to submit for publication.

## Results

**Figure 1:**
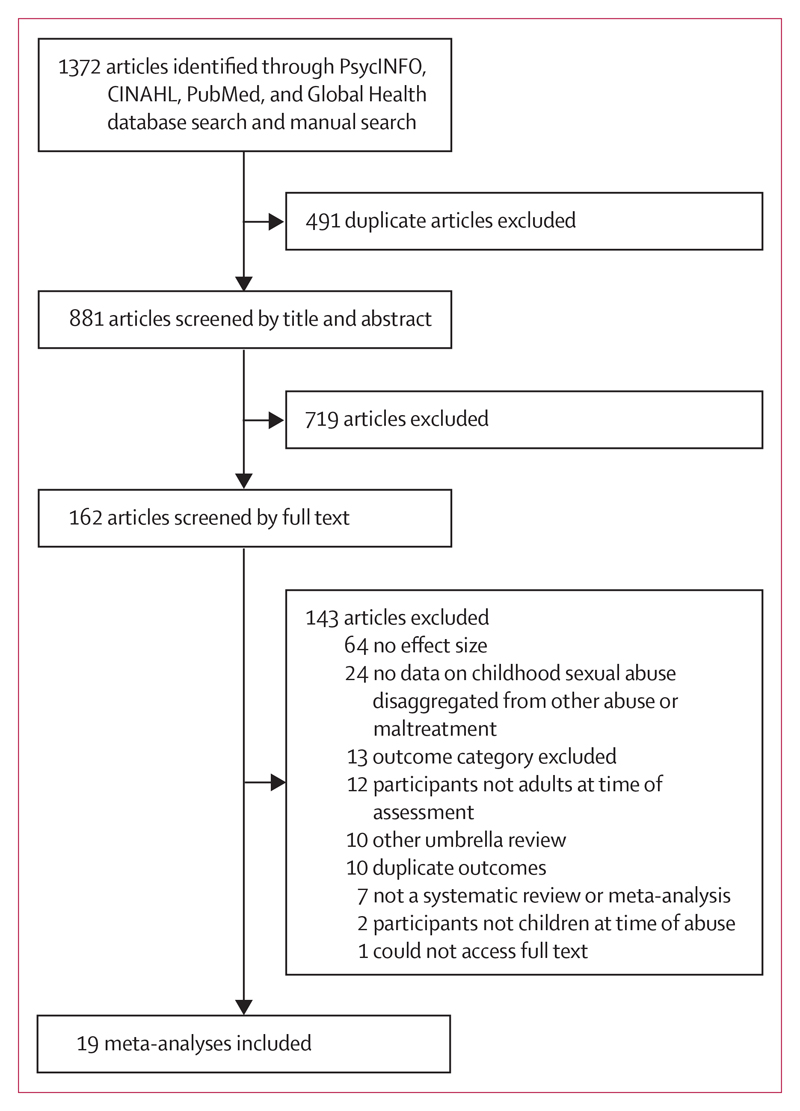
Study selection CINAHL=Cumulative Index to Nursing and Allied Health Literature.

The initial database and manual search yielded 1372 articles (figure 1). We identified 19 eligible meta-analyses with 4 089 547 participants across 28 outcomes and 559 primary studies (table 1).^2,3,5–9,43–54^ All meta-analyses were published between 1996 and 2018, and the primary studies included in these meta-analyses were reported from 1971 to 2017. The number of primary studies in each meta-analysis ranged from three to 62, and the number of participants ranged from 140 to more than 3 million.^6^ Outcomes included in the eligible meta-analyses were divided into psychosocial, psychiatric, and physical health outcomes, and childhood sexual abuse was associated with 26 of 28 specific outcomes.

**Figure 2:**
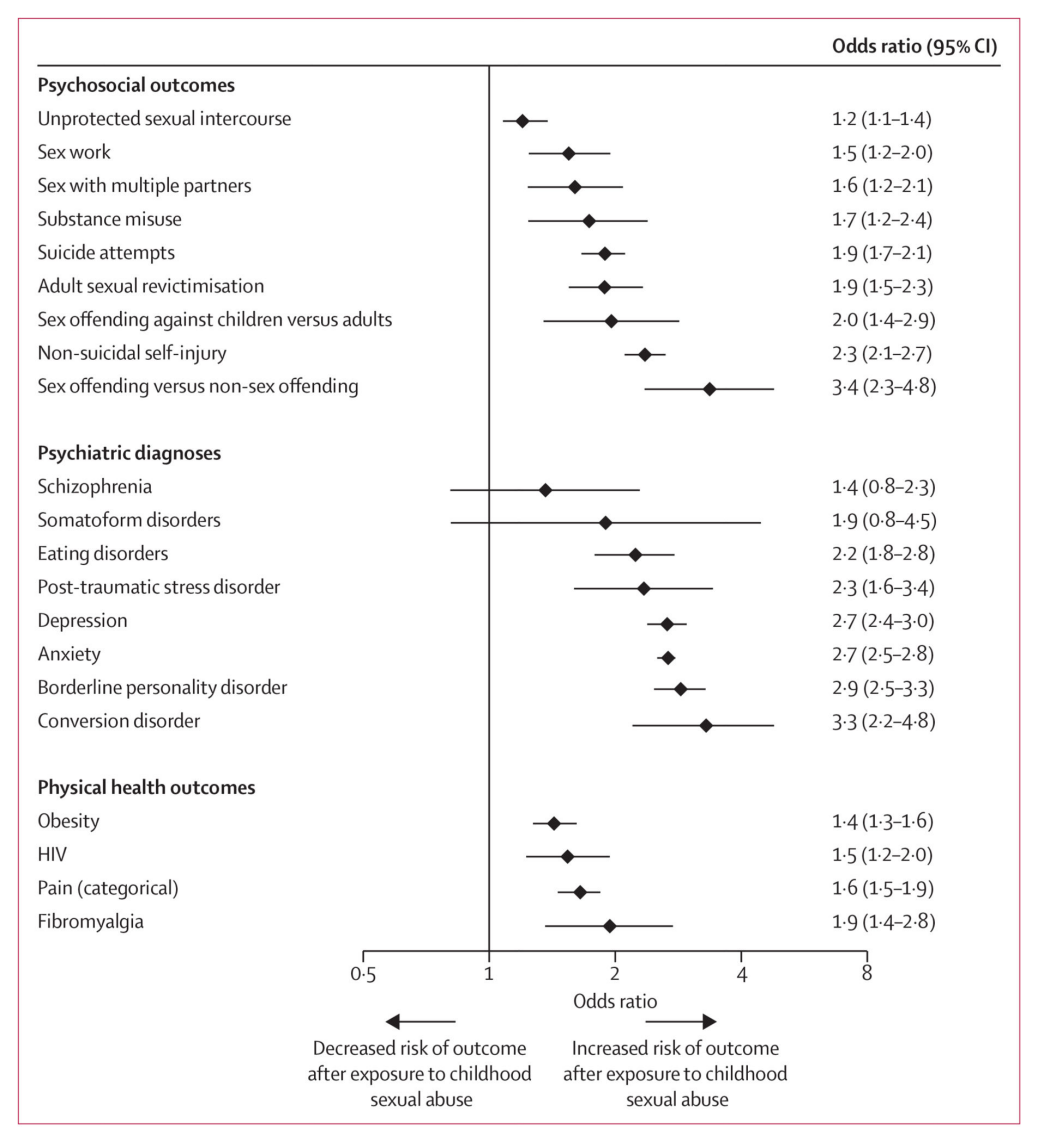
Risk estimates of long-term outcomes following childhood sexual abuse

The ORs mostly ranged from 2·2 (95% CI 1·8–2·8) to 3·3 (2·2–4·8) for psychiatric outcomes, from 1·2 (1·1–1·4) to 3·4 (2·3–4·8) for psychosocial outcomes, and from 1·4 (1·3–1·6) to 1·9 (1·4–2·8) for physical health outcomes (figure 2).

On the basis of thresholds for small, medium, and large effect sizes,^55^ childhood sexual abuse was associated with a small increased odds ratio (ie, less than 1·7) for seven outcomes and a medium odds ratio (ie, 1·7–3·5) for 21 outcomes, and was not associated with a large odds ratio (ie, more than 3·5) for any outcome (table 2). Outcomes with the strongest associations with childhood sexual abuse were sexual versus non-sexual offending (OR 3·4 [95% CI 2·3–4·8]), conversion disorder (3·3 [2·2–4·8]), borderline personality disorder (2·9 [2·5–3·3]), anxiety (2·7 [2·5–2·8]), and depression (2·7 [2·4–3·0]).

Population attributable fractions ranged from 1·7% (95% CI 0·7–3·3) for unprotected sexual intercourse to 14·4% (8·8–19·9) for conversion disorder. The highest population attributable fraction was 14·7% (9·6–19·9) for sexual offending versus non-sexual offending (table 2). However, these figures will likely be overestimated because the population attributable fractions were calculated using data without adjustment for confounders.

In studies with sufficient data, half of the studies had 95% prediction intervals that included the null OR of 1, more than half demonstrated evidence of excess significance, more than a fifth had small study effects, and more than a third had high heterogeneity (appendix pp 8–11).

In addition, systematic reviews of 15 of the 28 outcomes had low AMSTAR scores (appendix pp 6–9, 12). Systematic reviews of two outcomes (post-traumatic stress disorder and substance misuse) had high quality scores of 5 (out of 5), and another one (schizophrenia) received a score of 4 (appendix p 8). Quality was low for meta-analyses on both borderline personality disorder and anxiety (scoring 1 out of 5), and moderate (3 out of 5) for conversion disorder and sexual versus non-sexual offending.

For outcomes with high quality scores, we examined absolute rates reported in primary studies. Specifically, longitudinal cohort studies reported that among individuals who experienced childhood sexual abuse, 503 (28%) of 1809 developed substance misuse^56^ and 36 (38%) of 96 developed post-traumatic stress disorder.^57^

Among the included systematic reviews, a small number of studies explored sources of heterogeneity among primary studies. No differences were found on the effect of childhood sexual abuse by age on conversion disorders, fibromyalgia, and borderline personality disorders,^46,48,51^ by gender on depression,^53^ by study design on obesity and psychosis,^7,9^ and by adjustment for confounders such as psychiatric comorbidity on obesity and eating disorders.^9,52^ However, the risk of HIV was higher when individuals experienced sexual abuse in late than in early adolescence.^8^ In addition, the effects of childhood sexual abuse on obesity attenuated after adjustment for current depression,^9^ and the risk of anxiety after childhood sexual abuse was higher in females than in males.^5^

## Discussion

In this umbrella review of 28 long-term outcomes of childhood sexual abuse, we summarised the evidence from 19 meta-analyses, including more than 4 million participants from more than 500 primary studies. Childhood sexual abuse was associated with 26 of 28 examined outcomes, including a wide range of psychosocial, psychiatric, and physical health outcomes. Among the 26 outcomes that were significantly associated with childhood sexual abuse, only two were based on systematic reviews with high quality assessment scores: substance misuse and post-traumatic stress disorder.

Our findings underscore the need to better understand the mechanisms underlying the association between childhood sexual abuse and long-term outcomes. Some existing research points to biological mechanisms through which childhood abuse increases the risk of psychopathology and physical illness, such as through the hypothalamic–pituitary–adrenal axis or inflammation.^58,59^ Some outcomes might also be explained by psychosocial mechanisms, such as risky sexual behaviour and distorted body image.^60^ For example, childhood sexual abuse is associated with risky sexual behaviours,^61,62^ which could lead to HIV.^63^ The association between childhood sexual abuse and physical health problems might also be partially explained by mediating psychiatric factors. For example, the effect of childhood sexual abuse on obesity might be due to depression or certain eating disorders. Childhood sexual abuse is an important predictor of depression over the life course and depression is prospectively linked to obesity; however, it is also possible that effects of childhood sexual abuse on depression and eating disorders are triggered by obesity.^64–69^

Our review has highlighted research gaps. Primary work should capitalise on prospectively collected measures of childhood sexual abuse to minimise misclassification and recall bias. Future research should also adopt more comprehensive models to account for confounds (eg, other forms of abuse), use stronger designs and analysis (eg, prospective studies) to examine causal inference, and explore source of heterogeneity to identify protective and risk factors. Meta-analyses should ensure more accurate and systematic presentation of data and follow consensus guidelines to facilitate replicability. In addition, we found no meta-analyses on bipolar disorder, which has shown worse clinical outcomes when it occurs in combination with childhood maltreatment,^70^ obsessive compulsive disorder, or homelessness.

Our results underscore the need for effective interventions. So far, research on the primary prevention of childhood sexual abuse has focused primarily on school-based interventions and home-based parenting interventions. There is some evidence that school-based programmes aimed at helping children to recognise and report sexual abuse improve children’s knowledge and protective behaviours,^71^ although they are not developed to prevent adverse outcomes after sexual abuse. A growing body of research also suggests that early childhood home visit and parent education programmes promoting recognition of childhood sexual abuse and early symptoms of adverse outcome might prevent or reduce the risk of child maltreatment overall.^72–75^ In addition, physicians might play a part in screening for childhood sexual abuse, determining the need to report sexual abuse to appropriate safeguarding authorities, and coordinating care with other health professionals to prevent long-term poor outcomes.^76^

Research has indicated the effectiveness of interventions for post-traumatic stress disorder in individuals who have experienced childhood sexual abuse^77,78^ as well as depression.^78^ More specifically, research on various treatment modalities for individuals who have experienced childhood sexual abuse provides some support for cognitive behavioural interventions, particularly the efficacy of trauma-focused cognitive behavioural therapy for young people with post-traumatic stress disorder, anxiety, or depressive symptoms who have been sexually abused.^79–82^ However, research on treatments for some of the other important outcomes reported, particularly substance misuse and sexual revictimisation, in individuals who have been sexually abused is required. Another area for research is to improve translation of effective interventions into policy and practice, such as establishing community programmes to respond to sexual abuse. Furthermore, to prevent psychopathologies and other outcomes after sexual abuse, more research on the developmental mechanisms is necessary.

The sexual versus non-sexual offending finding does not mean an increased risk of offending in this population—rather, that if an individual has committed a crime, then there is an increased likelihood of being a sexual offender. By contrast, indicative absolute rates on two psychiatric outcomes with high quality scores were high, 28% for substance misuse and 38% for post-traumatic stress disorder, suggesting that interventions for these outcomes should be prioritised.

Strengths of this umbrella review include testing quantitative measures of research and outcome quality, allowing for the comparison of findings across outcomes, and the discrimination between higher and lower quality research findings. Another strength is the inclusion of a wide range of psychosocial, psychiatric, and physical health outcomes, given that many previous meta-analyses and umbrella reviews have focused on either a single or narrow subset of outcomes.^12–15^ Moreover, temporal ordering between the predictor and outcome of interest (ie, childhood sexual abuse occurring before 18 years of age and outcome measurement occurring after 18 years of age) was part of inclusion criteria, reducing the risk of conflating short-term effects of childhood abuse with adult consequences.

Several limitations should be noted. First, only a small number of the included systematic reviews explored sources of heterogeneity among primary studies by age,^46,48,51^ and study design.^7,9^ Other factors, including familial features (protective factors such as stable family environment and supportive relationships), characteristics of abuse, or overlap with other types of child abuse, are likely to moderate or mediate the association between sexual abuse and later outcomes, so not accounting for them might lead to overestimation of effect sizes.^83,84^ Future studies addressing these confounding factors are necessary for a more precise estimate of the link between child sexual abuse and later outcomes, including using family-based designs. In addition, collection of more detailed information about the nature of the abuse could help to disentangle the effects of different types of abuse. Second, the primary studies included in the meta-analyses were often based on retrospective recall of childhood sexual abuse by adults, and retrospective reports are suboptimal proxies for prospectively collected measures of childhood sexual abuse.^11^ Thus, future research with prospective designs is necessary. Apart from repeated measures of individuals at different timepoints with questionnaires,^85^ one other possibility could be using linked register-based datasets in which sexual abuse victimisation and physical and mental health outcomes are longitudinally recorded.^86,87^ Official register data would reduce report bias of sexual abuse and allow prospective studies of its links to later outcomes. Third, there is a high co-occurrence of childhood sexual abuse with other forms of child abuse, which is associated with poorer psychosocial and health outcomes.^88–90^ However, as a result of insufficient data, we were not able to take into account effects of other forms of child abuse in this umbrella review. Finally, our analyses showed that current evidence of the association between sexual abuse and health outcomes is inconsistent and studies with significant results were more likely to be published than those with non-significant findings. Future research is necessary to identify contributing factors and to decrease publication bias against studies with non-significant findings.

This umbrella review found that childhood sexual abuse is associated with elevated risks of long-term psychosocial, psychiatric, and physical health outcomes. In particular, there is high-quality evidence for associations between childhood sexual abuse and two psychiatric disorders (schizophrenia and post-traumatic stress disorder) and one psychosocial outcome (substance misuse). Because both relative risks and absolute rates for certain outcomes following childhood sexual abuse have been shown to be increased, this review suggests prioritising interventions that reduce the development of those outcomes that have a high-quality evidence base. Notable gaps include the need for further meta-analyses assessing outcomes for which the current review literature is low quality, and reviews on outcomes (bipolar disorder, obsessive-compulsive disorder, and homelessness) that currently lack systematic reviews. In addition, more empirical studies are necessary to clarify developmental pathways from sexual abuse to health-related and psychosocial outcomes, as well as treatment outcome research in individuals who have experienced childhood sexual abuse.

## Supplementary Material

Appendix

## Figures and Tables

**Table 1:** Characteristics of meta-analyses included in this umbrella review of outcomes following childhood sexual abuse

	Number of databases searched	Outcome	Number of primary studies	Sample size	Review (year range)	Country
Amado et al (2015)^5^	4	Anxiety disorder	62	93 075	1995–2014	Spain
Amado et al (2015)^5^	4	Anxiety symptoms	41	24 270	1995–2014	Spain
Amado et al (2015)^5^	4	Depressive symptoms	59	33 293	1988–2014	Spain
Arriola et al (2005)^2^	4	Adult sexual revictimisation	21	20 956	1975–2001	USA
Arriola et al (2005)^2^	4	Sex work	23	14 996	1975–2001	USA
Arriola et al (2005)^2^	4	Sex with multiple partners	23	16 560	1975–2001	USA
Arriola et al (2005)^2^	4	Unprotected sexual intercourse	16	11 770	1991–2001	USA
Chen et al (2010)^6^	9	Post-traumatic stress disorder	3	788	1999–2006	USA
Chen et al (2010)^6^	9	Schizophrenia	3	3 131 503	1997–2004	USA
Chen et al (2010)^6^	9	Sleep disorders	1	140	1988	USA
Chen et al (2010)^6^	9	Somatoform disorders	3	308	1997–2008	USA
Danese et al (2014)^9^	3	Obesity	26	161 195	NA	UK
Fossati et al (1999)^50^	2	Borderline personality disorder	21	2479	1987–1994	Italy
Halpern et al (2018)^51^	3	Substance misuse	7	22 527	1996–2017	Brazil
Hauser et al (2011)^52^	4	Fibromyalgia	10	1487	1995–2010	Germany
Irish et al (2010)^53^	3	Pain (continuous)	9	4934	1992–2007	USA
Irish et al (2010)^53^	3	Pain (categorical)	12	222 893	1992–2007	USA
Jespersen et al (2009)^3^	3	Sexual offending against children *vs* adults*	15	2296	1979–2003	Canada
Jespersen et al (2009)^3^	3	Sexual offending *vs* non-sexual offending	17	2798	1987–2003	Canada
Klonsky et al (2008)^54^	3	Non-suicidal self-injury	43	13 687	1980–2006	USA
Lloyd et al (2012)^8^	6	HIV	5	7796	1995–2009	USA
Ludwig et al (2018)^43^	2	Conversion disorder	15	2083	1971–2016	Germany
Molendijk et al (2017)^44^	3	Eating disorders	49	15 006	1985–2015	Netherlands
Nelson et al (2016)^45^	3	Depression	57	74 461	NA	Germany
Neumann et al (1996)^46^	1	Traumatic stress responses	4	NA	NA	USA
Ng et al (2018)^47^	5	Suicide attempts	47	151 476	1993–2017	Singapore
Quinones-Munoz (2001)^48^	3	Psychological symptoms	5	3720	1992–98	USA
Ulrich et al (2005)^49^	5	Somatisation	11	NA	NA	USA
Varese et al (2012)^7^	4	Psychosis	20	53 050	1984–2011	UK

NA=not available.

* Originally reported as “sexual offending against adults versus children”, so the effect size data included in the following analysis are the reciprocal of the data reported in the original meta-analysis.

**Table 2:** Effect of childhood sexual abuse on long-term outcomes, quality measurements, and population attributable fractions

	Odds ratio	95% CI	95% prediction interval	*I*^2^ (%)	95% CI for *I*^2^	Excess statistical significance ratio	Population attributable fraction (%)	95% CI for population attributable fraction
Sexual offending *vs* non-sexual offending	3·4	2·3–4·8	1·1–10·3	54%	21–74	··	14·7%	9·6–19·9
Conversion disorder	3·3	2·2–4·8	··	41%	0–68	··	14·4%	8·8–19·9
Borderline personality disorder	2·9	2·5–3·3	··	30%	0–59	··	12·4%	10·4–14·4
Anxiety	2·7	2·5–2·8	··	··	··	0·5	11·7%	7·8–15·8
Depression	2·7	2·4–3·0	··	65%	54–74	··	11·4%	9·8–12·9
Post-traumatic stress responses	2·6	2·2–2·9	··	··	··	··	10·9%	9·9–12·7
Psychosis	2·4	2·0–2·9	··	45%	7–67	1·4	9·8%	7·4–12·4
Non-suicidal self-injury	2·3	2·1–2·7	··	51%	32–66	1·4	9·7%	8·2–11·4
Post-traumatic stress disorder	2·3	1·6–3·4	1·0–5·4	0%	0–0	1·0	9·6%	4·8–15·0
Eating disorders	2·2	1·8–2·8	··	64%	51–73	··	9·0%	6·2–12·0
Anxiety symptomatology	2·0	1·9–2·0	··	··	··	1·5	7·3%	6·9–7·8
Depressive symptomatology	2·0	1·9–2·0	··	-	-	1·5	7·3%	6·9–7·8
Pain (continuous)	2·0	1·3–3·0	··	··	··	0·9	7·8%	2·6–13·1
Sexual offending against children *vs* adults	2·0	1·4–2·9	0·5–7·1	70%	50–83	1·7	7·3%	3·0–12·4
Fibromyalgia	1·9	1·4–2·8	1·0–3·8	20%	0–61	1·7	7·2%	3·0–11·8
Adult sexual revictimisation	1·9	1·5–2·3	··	94%	92–95	1·4	6·9%	4·5–9·7
Somatoform disorders	1·9	0·8–4·5	0·3–14·1	4%	0–26	1·9	6·9%	1·8–18·8
Suicide attempts	1·9	1·7–2·1	1·7–2·2	84%	80–88	0·9	6·9%	5·3–8·3
Psychological symptoms	1·7	1·5–2·0	0·7–4·3	77%	45–91	1·1	5·7%	4·0–7·8
Substance misuse	1·7	1·2–2·4	1·1–2·6	42%	0–75	0·9	5·8%	2·1–10·0
Pain (categorical)	1·6	1·5–1·9	··	··	··	1·3	5·2%	3·8–6·7
Sex with multiple partners	1·6	1·2–2·1	··	92%	90–94	1·4	4·8%	2·1–8·2
Somatisation	1·6	1·3–1·9	··	40%	0–7	··	4·5%	2·5–6·6
Sex work	1·5	1·2–2·0	··	76%	65–84	0·9	4·5%	2·1–7·3
HIV	1·5	1·2–2·0	0·9–2·8	50%	0–82	1·1	4·4%	1·9–7·2
Obesity	1·4	1·3–1·6	··	87%	82–91	1·1	3·6%	2·3–5·0
Schizophrenia	1·4	0·8–2·3	0·4–4·3	0%	0–0	0·9	3·0%	1·8–9·4
Unprotected sexual intercourse	1·2	1·1–1·4	··	51%	14–73	1·0	1·7%	0·7–3·3

Outcomes are ranked by descending effect size.

## Research in context

### Evidence before this study

We searched PsycINFO, PubMed, Cumulative Index to Nursing and Allied Health Literature, and Global Health for papers published from database inception to Dec 31, 2018, with no language restrictions. We used the search terms (“sexual abuse” OR “sexual assault” OR “sexual trauma” OR “sexual crime” OR “rape” OR “incest” OR “molestation” OR “victim*” OR “maltreatment”), AND (“child*” OR “youth” OR “adolescent” OR “young” OR “teen*”), AND (“meta-analy*” OR “meta-regression” OR “meta-synthesis”). We identified six umbrella reviews that primarily considered mental health outcomes (such as anxiety or depression) or a single outcome (such as suicide), which were published more than 5 years ago, lacked quantitative synthesis and quality assessment, and were mostly based on small sample sizes.

### Added value of this study

This umbrella review synthesised meta-analytical evidence on the association between childhood sexual abuse and a wide range of different outcomes. We found that childhood sexual abuse was associated with a wide range of adulthood psychiatric diagnoses, negative psychosocial outcomes, and physical health outcomes.

### Implications of all the available evidence

Although there were consistently strong links between childhood sexual abuse and many long-term health and psychosocial outcomes, the quality of the underlying research was limited by small study effects, excess significance, and high heterogeneity between studies. However, two psychiatric disorders (post-traumatic stress disorder and schizophrenia) and one psychosocial outcome (substance misuse) had a high-quality evidence base, and the available evidence suggests that interventions mitigating later substance misuse and post-traumatic stress disorder should be prioritised. Notable gaps in the review literature included links between childhood sexual abuse and bipolar disorder, obsessive-compulsive disorder, and homelessness.
