# Supplementary material for: Long-term outcomes of childhood sexual abuse: an umbrella review
Source: Lancet Psychiatry. Author manuscript; Available in PMC 2020 Feb 12. (PMC7015702; doi:10.1016/S2215-0366(19)30286-X)
Supplement: Appendix [file EMS85713-supplement-Appendix.pdf]

**Appendix: Supplemental Figures and Tables**

**Table S1:** Search terms used in targeted DALY searches

| DALY | Search Terms |
|---|---|
| Ischaemic heart disease | Ischaemic heart disease, coronary artery disease, coronary heart disease |
| Lower respiratory infections | Lower respiratory infection, pneumonia, lung abscess, acute bronchitis |
| Stroke | Stroke, transient ischaemic attack |
| Diarrhoeal diseases | Diarrhoeal disease, diarrhoea, dysentery, cholera |
| Chronic obstructive pulmonary disease | Chronic obstructive pulmonary disease, COPD, chronic bronchitis, emphysema |
| HIV/AIDS | HIV, AIDS, human immunodeficiency virus |
| Road injury | Road injury, traffic injury, road injuries, traffic injuries |
| Alcohol use disorder | Alcohol use, alcoholism, alcoholic |
| Schizophrenia | Schizophrenia, schizophrenic |
| Bipolar disorder | bipolar disorder, manic depression, mania |
| Dysthymia | Dysthymia, dysthymic disorder, mild depression, chronic depression |
| Autism or Asperger's syndrome | Autism, autistic, Asperger, ASD |
| ADHD and conduct disorder | Attention deficit hyperactivity disorder, ADHD, conduct disorder |

**Table S2:** Outcomes excluded from this umbrella review by reason for exclusion

| **Non-behavioural psychosocial outcomes** | **Behavioural outcomes with fewer primary studies than the ten selected** | **Health outcomes that are sub-categories of other diagnoses[a]** | **Health outcomes that are not diagnoses or symptoms associated with included diagnoses** |
|---|---|---|---|
| Alcohol problems | Anal intercourse | Depression and/or anxiety | Cardiopulmonary symptoms |
| Anger | Online offending compared with normative groups | Dysthymia | Dissociation |
| Combined hippocampal volume | Offline offending compared with normative groups | Generalized anxiety disorder | Dysmenorrhea |
| Hostility | Online sexual offending compared with offline sexual offending | Panic disorder | Dyspareunia |
| Interpersonal problems | Paedophilic versus non-paedophilic sexual offending against children | Paranoia | Gastrointestinal symptoms |
| Interpersonal sensitivity | Suicide | Phobia | General health |
| Left hippocampal volume | | Social phobia | General sequelae |
| Locus of control | | Specific phobia | Index of general symptoms |
| Overall adjustment | | | Non-cyclical chronic pelvic pain |
| Right hippocampal volume | | | Non-epileptic seizures |
| Self-concept impairment | | | Obsessive-compulsive symptomatology |
| Self-esteem | | | Obsessions and compulsions |

| | | | |
|---|---|---|---|
| Sexual adjustment | | | Psychological correlates |
| Sexual problems | | | Psychological symptoms |
| Social adjustment | | | Psychotic symptoms |

Note: [a]Outcomes that are a subcategory of a major diagnosis were excluded.

**Table S3:** Data extracted from all meta-analyses by category of data

| General information | Eligibility criteria | Methodology | Study results |
|---|---|---|---|
| First author | Meta-analysis | Outcomes studied | Number of participants with exposure |
| Running title | Systematic review | Whether inclusion criteria are stated | Number of participants without exposure |
| Journal of publication | Childhood sexual abuse disaggregated from other abuse | Number of studies | Number of participants with outcome |
| Year of publication | Average participant age at time of abuse < 18 | Number of participants | Number of participants without outcome |
| Date that coding was completed | Average participant age at time of assessment > 18 | Mean age of participants | Summary statistics reported |
| | Provides aggregated effect size data for health or psychosocial outcomes | Percentage of participants who are female | Summary statistic confidence intervals |
| | | Percentage of participants from clinical samples | Number of participants in largest primary study |
| | | Percentage of participants from general population | Effect size of largest primary study |
| | | Number of databases used | Confidence interval of largest primary study |
| | | Number of countries | Heterogeneity statistic |
| | | Year range of review | |
| | | Study design (prospective, retrospective, cross-sectional, or mixed) | |
| | | Quality assessment procedures used | |

**Table S4:** AMSTAR checklist results and scores*

| Study | Outcome(s) | Q1 | Q2 | Q3 | Q4 | Q5 | Q6 | Q7 | Q8 | Q9 | Q10 | Q11 | Aggregate Score |
|---|---|---|---|---|---|---|---|---|---|---|---|---|---|
| Amado, 2015 | Anxiety, anxiety symptoms, depressive symptoms | - | - | - | - | Yes | Yes | - | - | - | - | Yes | 3 |
| Arriola, 2005 | Adult sexual revictimisation, sex trading, sex with multiple partners, unprotected sexual intercourse | - | Yes | Yes | - | Yes | Yes | - | - | Yes | - | - | 5 |
| Chen, 2010 | Posttraumatic stress disorder, schizoprhenia, sleep disorders, somatoform disorders | Yes | Yes | - | Yes | Yes | - | Yes | Yes | Yes | Yes | Yes | 9 |
| Danese, 2014 | Obesity | Yes | - | - | - | Yes | Yes | Yes | Yes | Yes | Yes | Yes | 8 |
| Fossati, 1999 | Borderline personality disorder | - | - | - | - | Yes | - | - | - | Yes | - | - | 2 |
| Halpern, 2018 | Substance misuse | Yes | Yes | Yes | - | - | Yes | Yes | Yes | Yes | Yes | - | 8 |
| Hauser, 2011 | Fibromyalgia syndrome | Yes | Yes | | | Yes | Yes | Yes | Yes | Yes | Yes | Yes | 9 |
| Irish, 2010 | Pain (continuous), pain (categorical) | - | - | - | Yes | Yes | Yes | - | - | - | - | Yes | 4 |

| | | | | | | | | | | | | | |
|---|---|---|---|---|---|---|---|---|---|---|---|---|---|
| Jespersen, 2009 | Sexual offending against children versus adults, sexual offending versus non-sexual offending | - | - | Yes | Yes | Yes | Yes | - | - | Yes | Yes | Yes | 7 |
| Klonsky, 2009 | Non-suicidal self-injury | - | - | Yes | - | Yes | Yes | - | - | Yes | Yes | Yes | 6 |
| Lloyd, 2012 | HIV | - | Yes | Yes | Yes | Yes | Yes | Yes | Yes | Yes | - | - | 8 |
| Ludwig, 2018 | Conversion disorder | Yes | Yes | Yes | - | Yes | - | Yes | Yes | Yes | Yes | - | 8 |
| Molendijk, 2017 | Eating disorders | Yes | Yes | Yes | - | - | Yes | Yes | Yes | Yes | Yes | - | 9 |
| Nelson, 2016 | Depression | Yes | Yes | Yes | - | - | - | - | - | Yes | Yes | - | 5 |
| Neumann, 1996 | Post-traumatic stress responses | - | - | - | - | Yes | - | - | - | Yes | - | - | 2 |
| Ng, 2018 | Suicide attempts | Yes | - | Yes | - | - | Yes | - | - | Yes | Yes | - | 5 |
| Quinones-Munoz, 2001 | Psychological symptoms | - | - | - | Yes | Yes | Yes | - | - | Yes | - | - | 4 |
| Ulrich, 2005 | Somatization | - | - | Yes | Yes | Yes | - | - | - | Yes | - | - | 4 |
| Varese, 2012 | Psychosis | Yes | Yes | Yes | Yes | Yes | Yes | Yes | Yes | Yes | Yes | Yes | 11 |

*Note.* Q= Question. *See http://amstar.ca/docs/AMSTARguideline.pdf for a list of the AMSTAR questions. The criteria for question 5 (Was a list of studies included and excluded provided?) and 11 (Was the conflict of interest included?) were modified to reflect the included studies. For question 5, only a list of included, not excluded, studies were required, and for question 11, funding and support documentation was required only for the meta-analyses, not for each component primary study. One study included in this umbrella review[50] was a replication of a prior meta-analysis[93] and therefore did not conduct original literature searches or data extraction. For this reason, the original meta-analysis, which was not otherwise included in the analysis of this umbrella review, was coded to supply data for questions 1, 2, 3, and 4 of the AMSTAR checklist.

**Table S5:** Quality analyses for meta-analyses of long-term health outcomes following childhood sexual abuse

| Outcome | Prediction interval excludes null value | Heterogeneity | Evidence of excess statistical significance | Evidence of small study effects | AMSTAR | Overall score |
|---|---|---|---|---|---|---|
| Adult sexual revictimisation | - | High | Yes | - | Low | 0 |
| Anxiety | - | - | No | - | Low | 1 |
| Anxiety symptoms | - | - | Yes | - | Low | 0 |
| Borderline personality disorder | - | Low | - | - | Low | 1 |
| Conversion disorder | - | Low | - | No | High | 3 |
| Depression | - | High | No | No | Low | 2 |
| Depressive symptoms | - | - | Yes | - | Low | 0 |
| Eating disorders | - | High | - | No | High | 2 |
| Fibromyalgia | No | Low | Yes | No | High | 3 |
| HIV | No | High | Yes | - | High | 1 |
| Non-suicidal self-injury | - | High | Yes | Yes | High | 1 |
| Obesity | - | High | Yes | Yes | High | 1 |
| Pain (continuous) | - | - | No | - | Low | 1 |
| Pain (categorical) | - | - | Yes | - | Low | 0 |
| Posttraumatic stress disorder | Yes | Low | No | No | High | 5 |
| Psychological symptoms | No | High | Yes | | Low | 0 |
| Psychosis | | Low | Yes | No | High | 3 |
| Schizophrenia | No | Low | No | No | High | 4 |
| Sexual offending against children vs. adults | No | High | Yes | - | High | 1 |
| Sexual offending vs. | Yes | High | No | No | High | 3 |

| | | | | | | |
|---|---|---|---|---|---|---|
| non-sexual offending | | | | | | |
| Sex trading | - | High | No | - | Low | 1 |
| Sex with multiple partners | - | High | Yes | - | Low | 0 |
| Somatization | - | Low | - | - | Low | 1 |
| Somatoform disorders | No | Low | Yes | No | High | 3 |
| Substance misuse | Yes | Low | No | No | High | 5 |
| Suicide attempts | Yes | High | No | Yes | Low | 2 |
| Post-traumatic stress responses | - | - | - | - | Low | 0 |
| Unprotected sexual intercourse | - | High | No | - | Low | 1 |

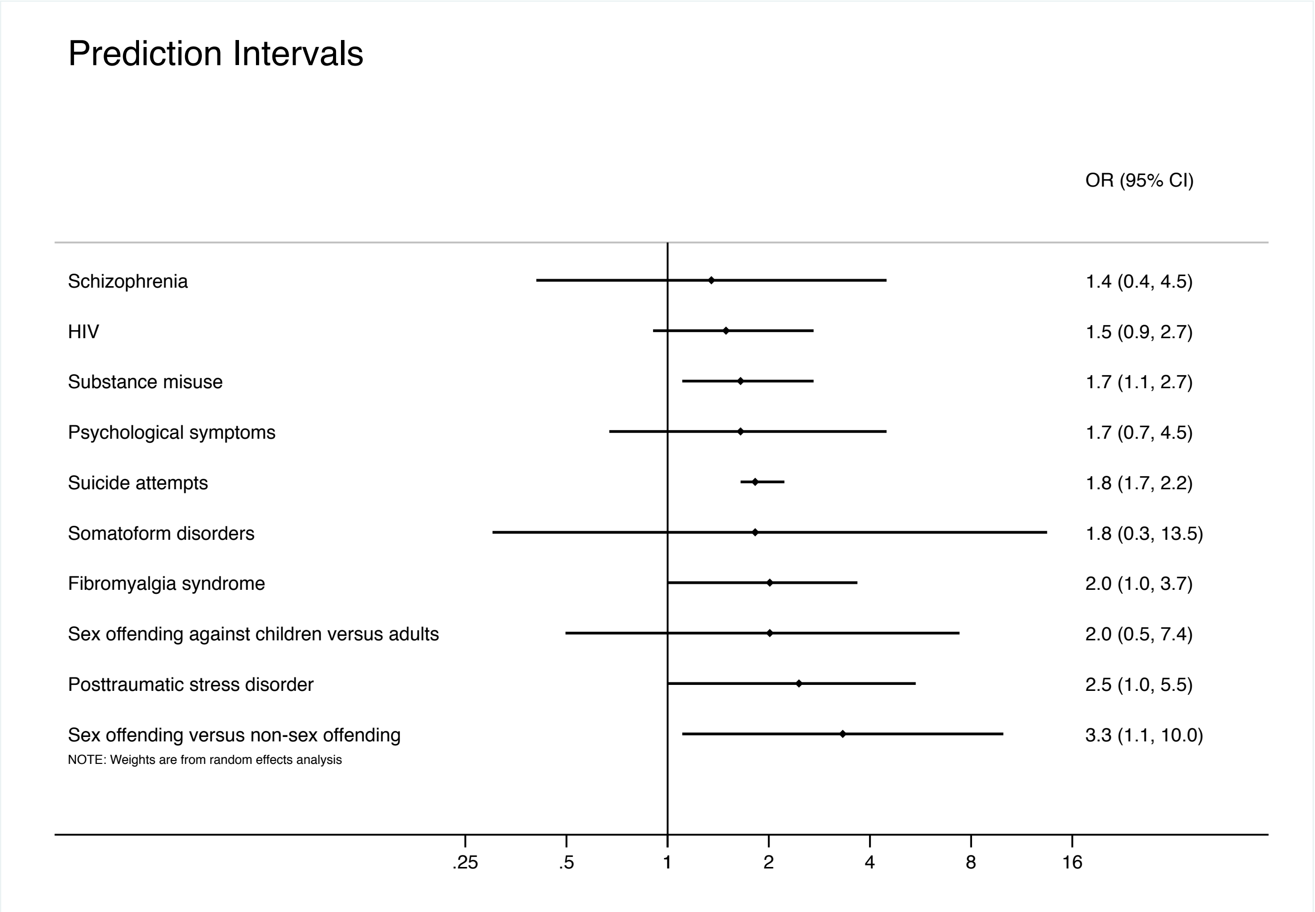


**Figure S1:** 95% prediction intervals for odds ratios [ORs] of long-term health outcomes following childhood sexual abuse

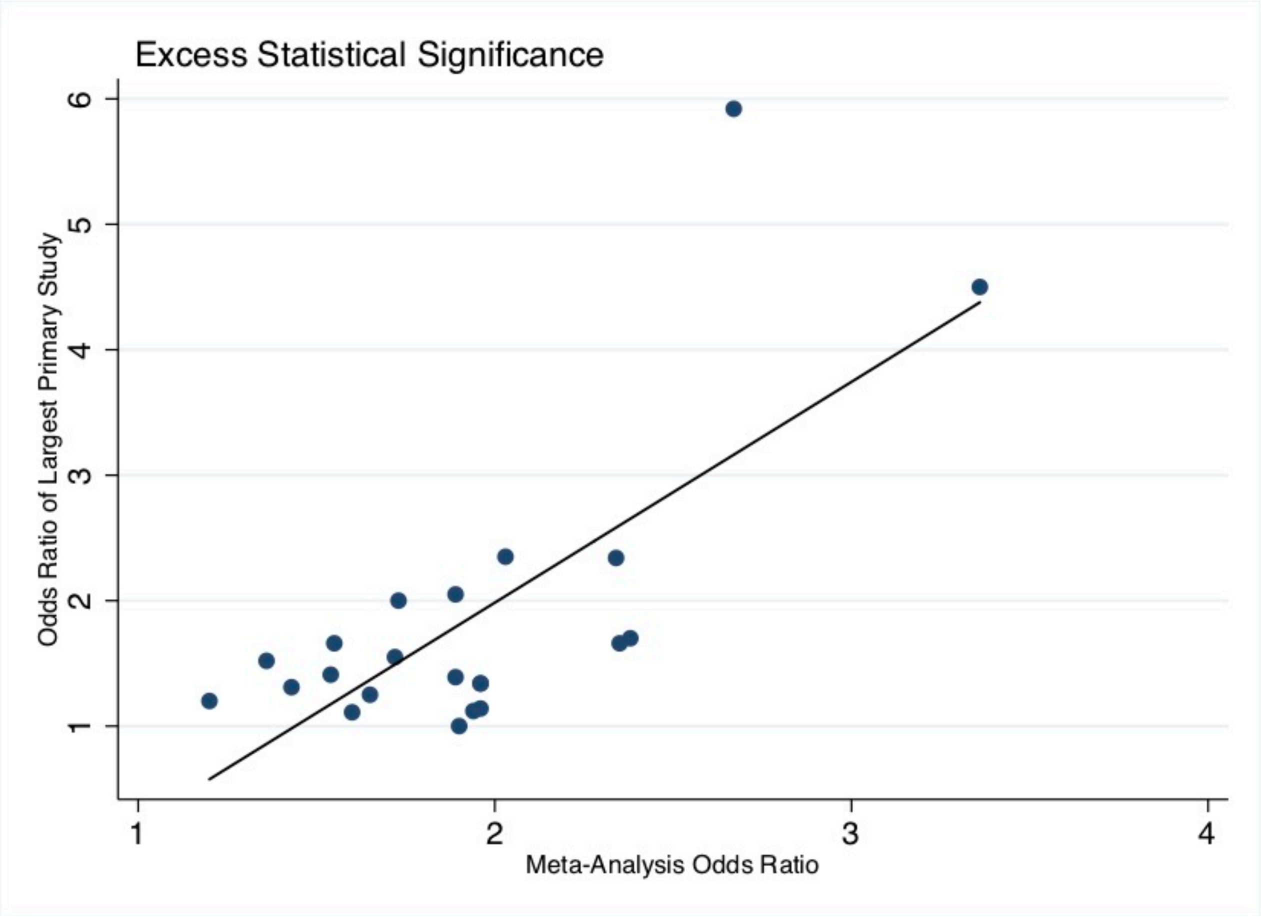


**Figure S2:** Odds ratios reported in the meta-analysis compared with largest primary study to test excess statistical significance

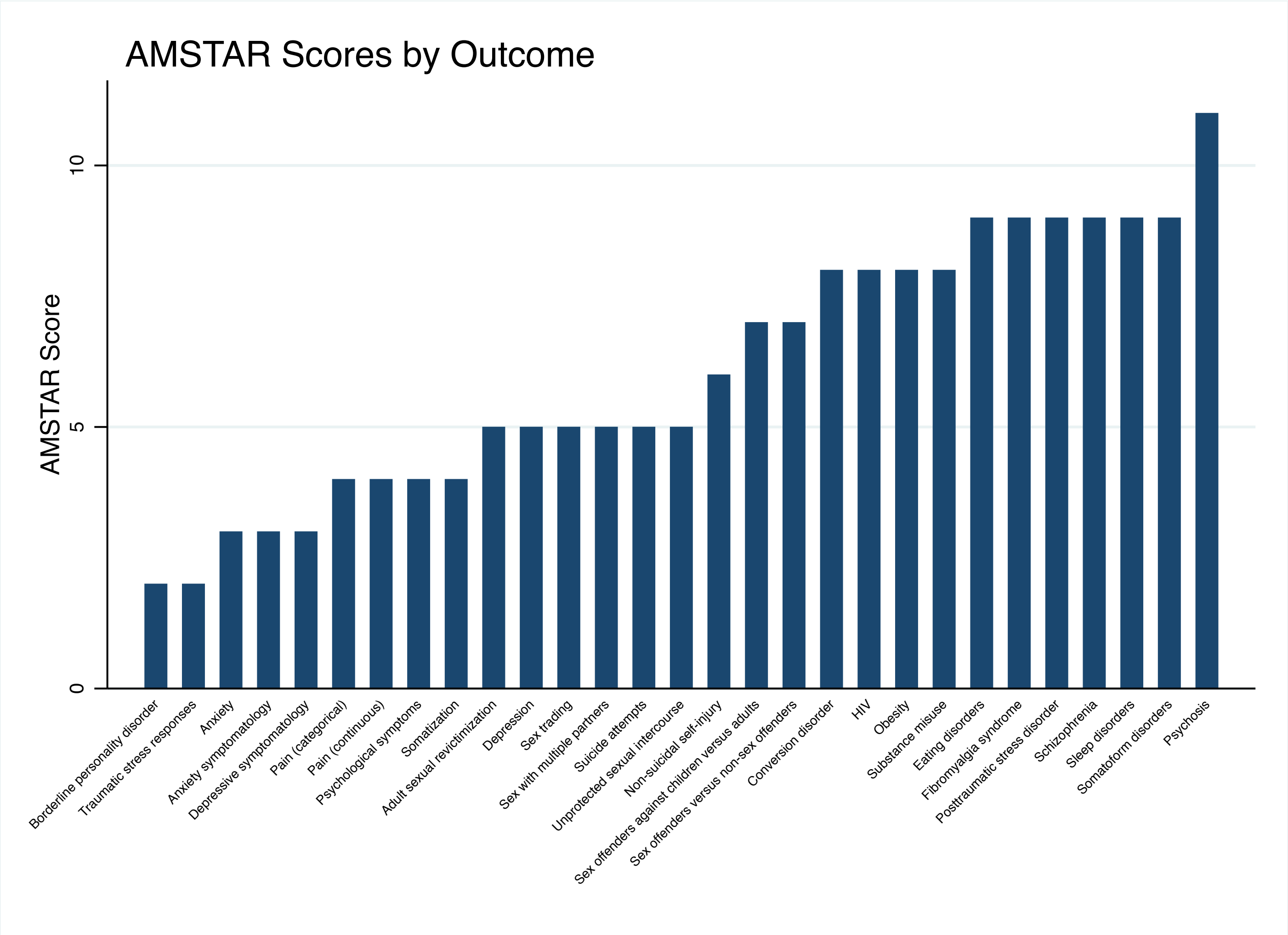


**Figure S3:** AMSTAR scores by long-term health outcome
